# The Regulatory Effect of Braided Silk Fiber Skeletons with Differential Porosities on In Vivo Vascular Tissue Regeneration and Long-Term Patency

**DOI:** 10.34133/2022/9825237

**Published:** 2022-11-11

**Authors:** Xili Ding, Weirong Zhang, Peng Xu, Wentao Feng, Xiaokai Tang, Xianda Yang, Lizhen Wang, Linhao Li, Yan Huang, Jing Ji, Diansheng Chen, Haifeng Liu, Yubo Fan

**Affiliations:** ^1^School of Engineering Medicine, Beihang University, Beijing 100083, China; ^2^Key Laboratory of Biomechanics and Mechanobiology (Beihang University), Ministry of Education, Beijing Advanced Innovation Center for Biomedical Engineering, School of Biological Science and Medical Engineering, Beihang University, Beijing 100083, China; ^3^eRobot Institute, School of Mechanical Engineering and Automation, Beihang University, Beijing 100083, China

## Abstract

The development of small-diameter vascular grafts that can meet the long-term patency required for implementation in clinical practice presents a key challenge to the research field. Although techniques such as the braiding of scaffolds can offer a tunable platform for fabricating vascular grafts, the effects of braided silk fiber skeletons on the porosity, remodeling, and patency in vivo have not been thoroughly investigated. Here, we used finite element analysis of simulated deformation and compliance to design vascular grafts comprised of braided silk fiber skeletons with three different degrees of porosity. Following the synthesis of low-, medium-, and high-porosity silk fiber skeletons, we coated them with hemocompatible sulfated silk fibroin sponges and then evaluated the mechanical and biological functions of the resultant silk tubes with different porosities. Our data showed that high-porosity grafts exhibited higher elastic moduli and compliance but lower suture retention strength, which contrasted with low-porosity grafts. Medium-porosity grafts offered a favorable balance of mechanical properties. Short-term in vivo implantation in rats indicated that porosity served as an effective means to regulate blood leakage, cell infiltration, and neointima formation. High-porosity grafts were susceptible to blood leakage, while low-porosity grafts hindered graft cellularization and tended to induce intimal hyperplasia. Medium-porosity grafts closely mimicked the biomechanical behaviors of native blood vessels and facilitated vascular smooth muscle layer regeneration and polarization of infiltrated macrophages to the M2 phenotype. Due to their superior performance and lack of occlusion, the medium-porosity vascular grafts were evaluated in long-term (24-months) in vivo implantation. The medium-porosity grafts regenerated the vascular smooth muscle cell layers and collagen extracellular matrix, which were circumferentially aligned and resembled the native artery. Furthermore, the formed neoarteries pulsed synchronously with the adjacent native artery and demonstrated contractile function. Overall, our study underscores the importance of braided silk fiber skeleton porosity on long-term vascular graft performance and will help to guide the design of next-generation vascular grafts.

## 1. Introduction

Cardiovascular disease caused by the global epidemic of arteriosclerosis is the main reason for disability and death worldwide [1, 2]. Autologous vessels, including the saphenous vein or mammary artery, are usually used for repairing small-diameter blood vessels (inner diameter < 6 mm) [3]. However, autologous grafts are unavailable due to donor site morbidity, vascular disease, or anatomic abnormalities [4]. Artificial vascular grafts with larger diameters have been successfully applied clinically for blood vessel replacement. However, small-diameter vascular grafts cannot be successfully used in clinical practice [5].

To achieve the mechanical properties and microstructure of natural artery, textile technique has been used to fabricate vascular grafts [6]. It allows certain degrees of decoupling of the chemical and mechanical properties of vascular grafts, enabling a wide range of mechanical properties using the same polymer yarn. Particularly, braiding provides a platform for fabricating small-diameter vascular grafts, which offers enhanced mechanical properties under various loading conditions, good flexibility, and structural stability, as well as controlled tissue regeneration [7, 8]. The chemical, mechanical, and morphometric properties of the braided vascular grafts can be adjusted to modulate the degree of induced inflammation and cell infiltration, with some parameters including polymer chemistry, fiber size and alignment, pore size, porosity, and degradation rate [9]. Among these parameters, porosity is found to be a key issue for the mechanical properties of braided vascular grafts, which can control cellular penetration and proliferation and affect the mechanobiologically mediated extracellular matrix production and remodeling [6].

The ideal regenerated graft should also have vascular smooth muscle cells (VSMCs) with contractile function, which can induce contraction of the vascular wall [10]. Wang et al. reported that one year after implantation, the contraction response of the neoartery was only 1–2% of that of the native artery, which indicated that the contractile function of the VSMCs was very low [11]. In another study, VSMCs oriented in the circumferential direction were regenerated after 3 months of implantation. After 18 months, the contractile function of regenerated VSMCs almost disappeared [12]. It has been demonstrated that biomechanical stimulation could modulate vascular remodeling and promote the contractile phenotype of VSMCs [13, 14]. Smooth muscle cells within the arterial wall are usually mechanically deformed in the form of periodic stretches generated by pulse waves. Such periodic dynamic forces are responsible for the morphology and function of VSMCs [15]. Therefore, we hypothesized that the braiding of silk fibers into a vascular graft will provide ideal mechanical properties that can subject VSMCs to continuous dynamic forces and improve the contractile function of the neoartery.

Degradable synthetic biomaterials such as polycaprolactone, polylactide-coglycolide, poly-L-lactic acid, and polyglycolic acid have been reported to generate vascular grafts and achieved promising results [16]. However, these synthetic biomaterials are not suitable for fabricating small-diameter vascular grafts because of thrombus formation and compliance mismatch [17]. Compared with synthetic biomaterials, natural biomaterials such as silk fibroin, elastin, and collagen have superior biocompatibility and cytocompatibility as a result of their functional molecules and embedded structure [18, 19]. Silk fiber has been used as a suture material in surgery for more than 2000 years [20, 21]. Silk is composed of silk fibroin and sericin. Silk sericin is considered to be of high immunogenicity associated with native silk fibers [22], while silk fibroin possesses excellent biocompatibility and low antigenicity [23, 24]. Silk fibroin was chosen for the current study due to its suitable mechanical properties, controllable biodegradability, minimal inflammation, and high processing ability to form various forms of scaffolds such as tubes, fibers, films, and porous sponges [25–28]. Different methods have been used to fabricate silk-derived grafts, resulting in tubes with various mechanical characteristics [29–32]. In addition, anticoagulant properties and inflammatory responses should also be considered during the design process [33–36]. Our previous work has shown that sulfated silk fibroin can increase the anticoagulant properties of vascular grafts and maintain the vascular cell phenotype [37]. In this study, we used poly(ethylene glycol) diglycidyl ether (PGDE) as a pore inducer for the silk fibroin solution to generate a sponge coating for braided vascular grafts [30, 38]. The silk tubes were generated using sulfated silk fibroin sponges. The tubes had different porosities and were evaluated mechanically. Grafts with different porosities were implanted into the rat abdominal aorta for various characterizations. In addition, the long-term performance of the braided vascular grafts was also investigated in this study.

## 2. Results

### 2.1. Finite Element Analysis

**Figure 1:**
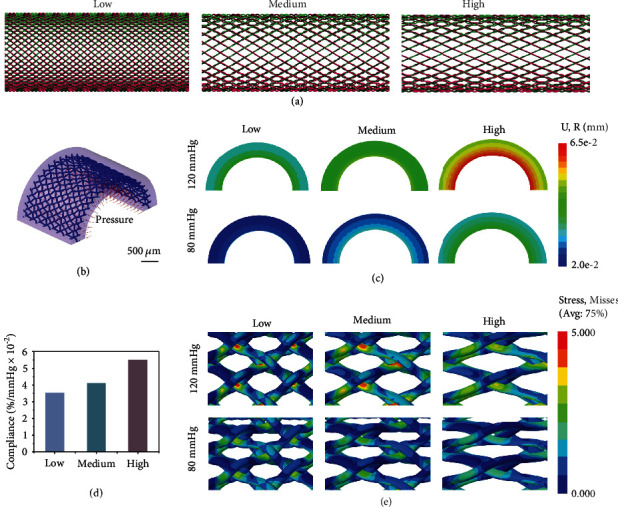
Finite element analysis. (a) Model of the different braided vascular grafts (low, medium, and high porosity). (b) Model of the braided silk fibroin vascular grafts and loading conditions of the simulation. (c) Cross-sectional views showing the deformation distribution of the grafts along the radius under different pressures. (d) The predicted compliance values of the different grafts obtained by the finite element analysis. (e) The maximum stress distribution of the braided tubes in the vascular grafts under different pressures. Scale bar: (b) 500 *μ*m.

An analytical model was made to further study the relationship between graft compliance and porosity of the braided tubes. As shown in Figure 1(a), three vascular graft models were developed with different porosities (low, medium, and high). Figure 1(b) shows the model of the braided silk fibroin vascular grafts and the loading conditions used in the simulation. Figure 1(c) shows the cross-sectional view of the deformation distribution of the vascular grafts with different porosities along the radial direction. For the same blood pressure, the radial deformation of the vascular grafts increased as the porosity of the braided tubes increased. The compliance values of the vascular grafts made from low-, medium-, and high-porosity tubes were 3.54%, 4.13%, and 5.49%, respectively (Figure 1(d)). Finite element analysis (FEA) showed that the compliance of the braided vascular grafts could be modulated by changing the porosity, and increasing the porosity resulted in a graft with greater compliance. The FEA model indicated that compliance could be obtained above 5%/mmHg × 10^−2^ by adjusting the porosity, which exceeded the compliance of saphenous vein (4%/mmHg × 10^−2^), the gold standard [29]. In addition, as shown in Figure 1(e), silk fibers were the main stress-bearing structure, and the position where two silk fibers crossed was the stress concentration area.

### 2.2. Mechanical Properties and Morphology of the Vascular Grafts

**Figure 2:**
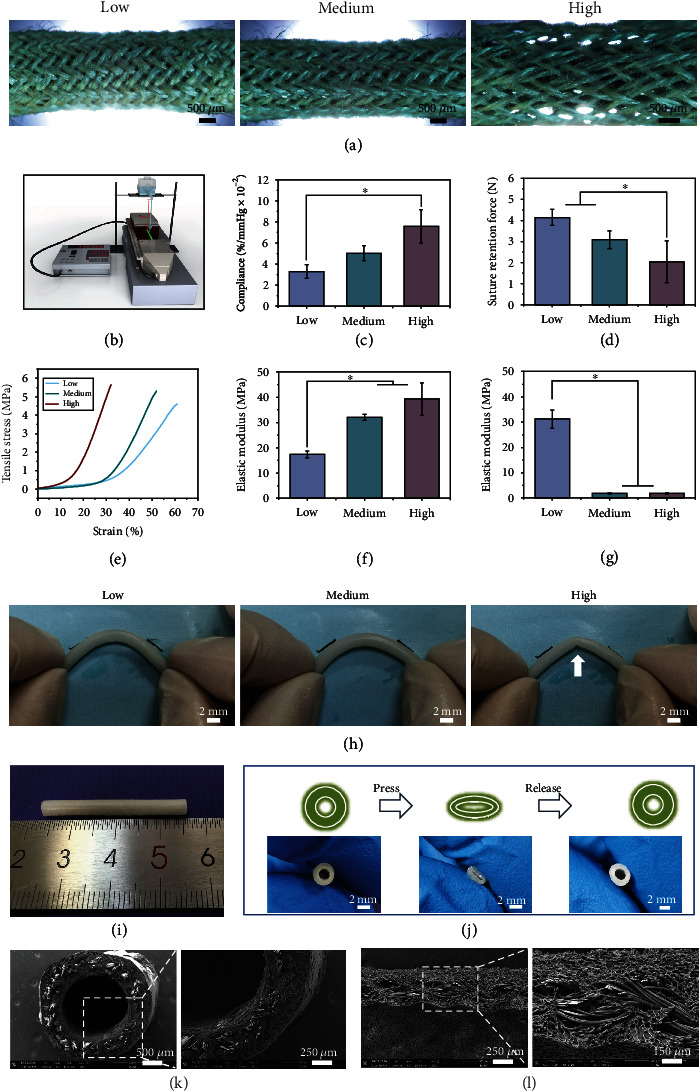
Mechanical and structural characterization of the vascular grafts. (a) Braided tubes with low, medium, and high porosity without a silk fibroin sponge layer. (b) Illustration of the experimental compliance measurement system. (c) Experimental compliance values of the different grafts. ^∗^*p* < 0.05. Data are presented as the mean ± SD (*n* = 3). (d) Suture retention force of the different grafts. ^∗^*p* < 0.05. Data are presented as the mean ± SD (*n* = 4). (e) Representative stress-strain curves of different grafts. (f) Longitudinal elastic modulus of the different grafts. ^∗^*p* < 0.05. Data are presented as the mean ± SD (*n* = 3). (g) Circumferential elastic modulus of the different grafts. ^∗^*p* < 0.05. Data are presented as the mean ± SD (*n* = 5). (h) Presentation of the antikink properties of the different grafts when the grafts were folded at 105°. The arrow indicates the kink in the high-porosity grafts. (i) Representative picture of the medium-porosity vascular graft. (j) Pictures showing that the graft retains its original shape after removal of the force. (k) SEM images of cross-sections of the grafts. (l) SEM images showing the braided fibers and the microstructure of the grafts.

The compliance of vascular grafts with different porosities was tested through experiments to verify the computational model. The compliance of the vascular grafts made from low-, medium-, and high-porosity tubes (Figure 2(a)) was tested using a self-designed compliance measurement system (Figure 2(b)). The compliance values of the vascular grafts made from low-, medium-, and high-porosity tubes increased from 3.3 ± 0.6%/mmHg × 10^−2^ to 7.6 ± 1.6%/mmHg × 10^−2^ (Figure 2(c)). The trend of the experimental compliance value is consistent with the computational results. The grafts with higher porosity showed higher compliance. The compliance value of the medium-porosity group was 5.0 ± 0.7%/mmHg × 10^−2^, which indicated that the compliance levels of vascular grafts can be adjusted by tailoring the porosity of the braided tubes [29].

The suture retention strength of the grafts decreased from 4.1 ± 0.4 N to 2.0 ± 1.0 N with increasing graft porosity (Figure 2(d)). The suture retention strength of the high-porosity group was lower than the good standard of suture retention strength for small-diameter vascular grafts (~2.5-3.0 N) [39], which indicated that the fibers in the high-porosity group could not protect the suture from loosening at the edges during the implantation process. Compared to low-porosity vascular grafts, the medium- and high-porosity groups had markedly increased in elastic modulus and maximum stress (Figure 2(e)). In addition, the longitudinal elastic modulus of the vascular grafts increased from 17.4 ± 1.3 MPa to 39.3 ± 6.5 MPa, but the medium- and high-porosity groups showed no significant difference (Figure 2(f)). Figure 2(g) shows that the circumferential elastic modulus of the vascular grafts decreased from 31.0 ± 3.6 MPa to 1.8 ± 0.2 MPa. Furthermore, the fibers in the low- and medium-porosity groups could prevent the development of an unfavorable vascular kink, while the high-porosity group could form a dead fold (Figure 2(h)). Taken consideration of the compliance, suture retention strength, and antikink properties, the medium-porosity group was chosen for morphology observation.

The medium-porosity vascular graft sample was shown in Figure 2(i). Figure 2(j) shows that the obtained vascular grafts had good structural recoverability after being pressed. The three layers of the grafts showed no visible separation (Figure 2(k)). Micropores could be observed in the external and internal layers (Figure 2(l) and Figure S1). The thickness of the graft was 534.05 ± 36.67 *μ*m. The size of the pores on the inner surface was 28.7 ± 7.8 *μ*m. In addition, SEM images showed that the micropores in the wall of the graft were highly interconnected (Figure 2(l)), which could facilitate cell infiltration.

### 2.3. In Vivo Performance of Vascular Grafts with Different Porosities

The *in vivo* performance of the vascular grafts with different porosities was evaluated with a rat abdominal aorta model. Five rats were used for the low-porosity group, 5 rats were used for the medium-porosity group, and 3 rats were used for the high-porosity group. Three animals in the high-porosity group died of bleeding, indicating that preventive measures should be taken in the early stage after implantation in the high-porosity group. There was only one rat in the low-porosity group that was patent, and this rat was recovered at 15 days. After 15 days or one month, the remaining 4 rats in the low-porosity group were retrieved and showed blocked grafts (Figure S2).

**Figure 3:**
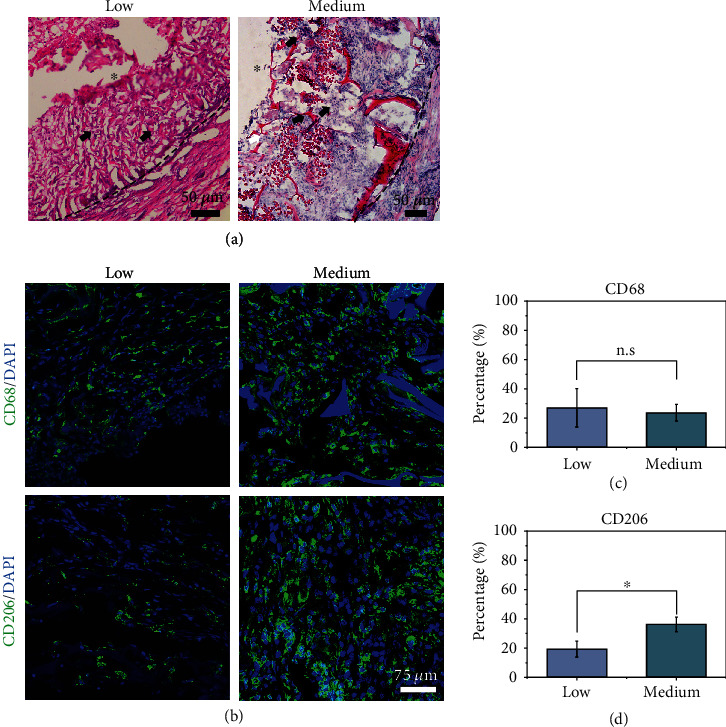
Cellularization and inflammatory response in vascular grafts with low and medium porosity. (a) H&E staining shows cellularization of the low and medium-porosity vascular grafts at 15 days. (b) Immunostaining of the macrophage marker CD68 and M2 macrophage marker CD206 at 15 days. (c, d) Percentage of CD68^+^ and CD206^+^ cells of the regenerated graft at 15 days. ^∗^*p* < 0.05. Data are presented as the mean ± SD (*n* = 3). The black arrows indicate that the cells infiltrated into the vascular wall. The black dashed lines indicate the edges of the graft. Lumen is marked with ^∗^.

Cell infiltration was evaluated by H&E staining. In the low-porosity vascular grafts, cells only slightly penetrated the graft wall, and the braided silk fiber easily detached from the glass slide during H&E staining due to fewer infiltrated cells within the braided silk fiber layer (Figure 3(a) and Figure S3).

Macrophages play a key role in the patency and remodeling of vascular grafts. There was no significant difference in the number of CD68^+^ macrophages between the low- and medium-porosity vascular grafts at 15 days (Figures 3(b) and 3(c)). Macrophages can be induced into M2 or M1 macrophages. The polarized shift of macrophages to M2 is a promising strategy to promote vascular graft remodeling [35]. Compared with the low-porosity vascular grafts, the medium-porosity vascular grafts had more CD206^+^ macrophages (M2) within the graft wall, suggesting the constructive remodeling of grafts (Figures 3(b) and 3(d)).

The distribution of macrophages in the medium-porosity vascular grafts was also assessed at different time points. As shown in Figure S4A-D, a large number of CD68^+^ cells were found within the graft wall. The number of CD68^+^ continuously decreased over time. The distribution of CD68-positive cells tended to change to the outside of the graft wall. Real-time RT-PCR data showed that the mRNA expression levels of the M1 macrophage marker iNOS decreased over time, while the mRNA expression levels of the M2 macrophage marker CD206 significantly increased (Figure S4E).

### 2.4. Patency and Endothelialization of the Medium-Porosity Vascular Grafts

**Figure 4:**
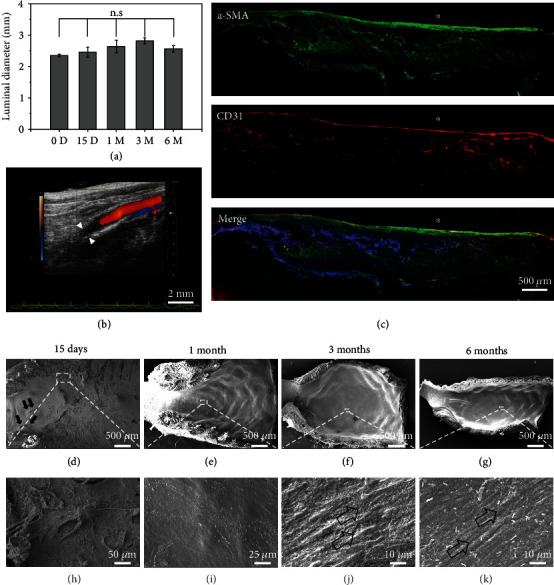
*In vivo* evaluation of medium-porosity vascular grafts. (a) The luminal diameter of the grafts was evaluated over time. Data are presented as the mean ± SD (n = 4). “n.s.” means no significance. (b) Patency of the vascular grafts 6 months after implantation was evaluated by the color Doppler ultrasound imaging system. Arrows indicate anastomosis. (c) Immunofluorescence staining showed the distribution of endothelial cells (CD31, red) and VSMCs (*α*-SMA, green) in the regenerated graft at 3 months. (d–k) SEM images of the lumen of the vascular grafts at different time points. Lumen is marked with ^∗^. Black arrows in (d) indicate the suture. Arrows in (j) and (k) indicate that the endothelial cells were arranged in the direction of blood flow.

Due to the high mortality and low patency of high- and low-porosity vascular grafts, only the medium-porosity vascular grafts were used for different time point evaluations (15 days, 1, 3, 6, and 24 months). A total of 28 rats were used to evaluate the performance of the medium-porosity vascular grafts. The patency rates for 15 days and 1, 3, and 6 months were 100% (6/6), 100% (6/6), 100% (6/6), and 100% (6/6), respectively. The diameter of the lumen did not change over the implantation time, which indicates that there were no aneurysms or restenosis within the grafts (Figure 4(a)). In addition, microvessels were visible in the surrounding tissue around the grafts, which is indicative of vascularization (Figure S5A). Observed under a stereomicroscope, there was no thrombus or intimal hyperplasia in the lumen of all the regenerated grafts (Figure S5B). Doppler ultrasound was used to assess the patency of the regenerated grafts, and all the grafts were patent at different time points. Figure 4(b) and Figure S6 show that there was no thrombosis or restenosis in the grafts 6 months after implantation.

A confluent endothelial monolayer and excellent neoartery regeneration were observed by costaining VSMCs and endothelial cells (Figure 4(c)). Furthermore, the lumen of the grafts was examined by SEM. SEM images showed that there were no thrombosis or platelet aggregates in the lumen at 15 days, and cell layers could only be found in the area near the suture (Figure 4(d)). Furthermore, along with the graft, part of the luminal surface of the grafts was not covered by cells, but there were no platelet aggregates or thrombosis formations in the area where the graft material could still be visualized (Figures 4(d) and 4(h)). However, the cells covered the entire luminal side of the graft one month after implantation (Figures 4(e) and 4(i)). After 3 months, complete endothelialization was achieved as indicated by staining with CD31 antibody, which further confirmed the formation of the endothelial layer (Figure 4(c)). In addition, after 3 months, the endothelial cells were arranged in the direction of the blood flow, and the arrangement was similar to the natural endothelial cells (Figures 4(j) and 4(k)).

### 2.5. Vascular Regeneration and Remodeling In Vivo

**Figure 5:**
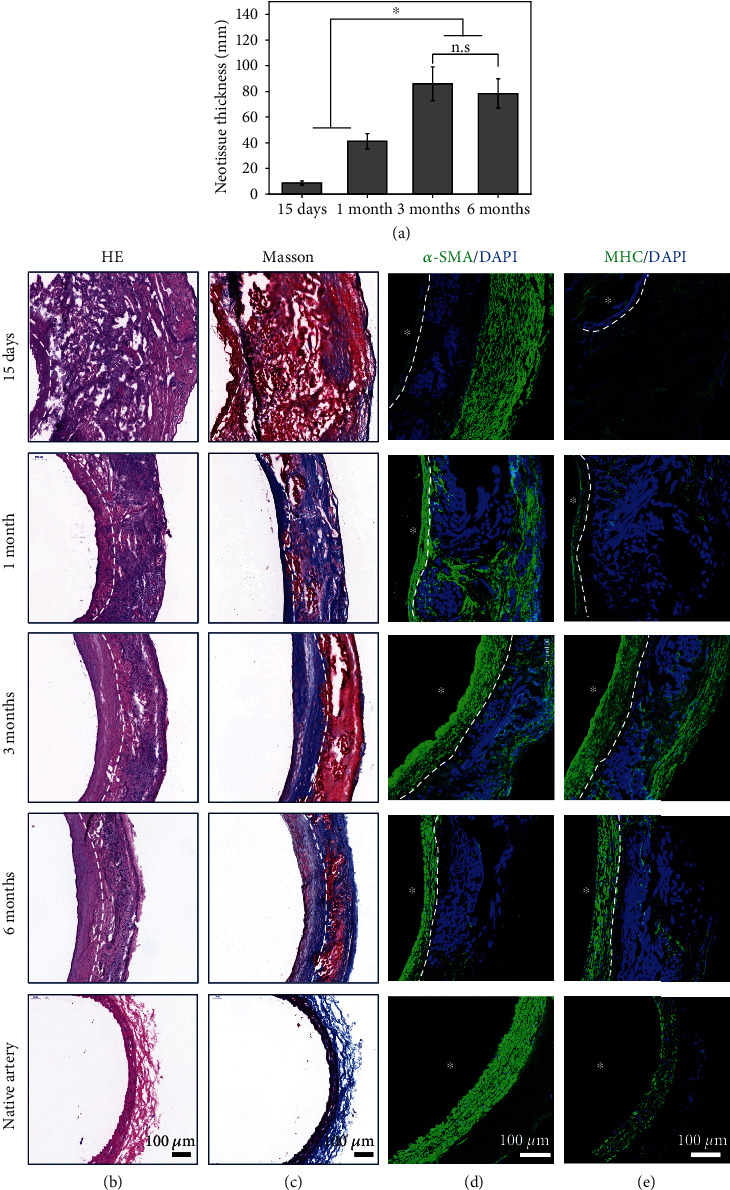
Histological analysis of the regenerated medium-porosity grafts at different time points in comparison with the native artery. (a) The neotissue thickness changed during the vascular development process. ^∗^*p* < 0.05. Data are presented as the mean ± SD (*n* = 5). “n.s.” means no significance. (b) H&E staining of the regenerated grafts at each time point and the native artery. The native abdominal aorta of rats without operation was used as the control group. (c) Masson's trichrome staining of the regenerated grafts at each time point and the native artery. (d, e) Regeneration of VSMCs with *α*-SMA antibody and MHC antibody staining. Lumen is marked with ^∗^. The white dotted line denoted the interface between neotissue and the original graft.

H&E and immunofluorescent staining were used to evaluate VSMC regeneration of the medium-porosity vascular grafts after implantation. The regenerated neotissue thickness increased during the vascular regeneration process (Figure 5(a)). H&E staining at 15 days suggested that the graft wall pores were completely occupied by the host cells, indicating that the pore structure facilitated the rapid infiltration of cells and collagen remodeling (Figures 5(b) and 5(c)). Figure 5(d) shows that the cells positive for *α*-SMA were mainly distributed outside of the grafts. The cells expressing *α*-SMA inside the grafts increased over time. Mature VSMCs have both contractile and synthetic phenotypes. VSMCs with a contractile phenotype secrete contractile proteins such as *α*-SMA, MHC, and CNN-1 [40, 41]. Few cells expressed MHC at an early stage, but a large number of cells expressed MHC at 3 months and 6 months, which indicated that the SMC phenotype turned toward a contractile state with high expression of late-stage (MHC) contractile proteins (Figure 5(e)). Real-time RT-PCR data showed that the mRNA expression level of MHC at 3 months was higher than that at 15 days and 1 month (Figure S7). The vascular smooth muscle layer is crucial for maintaining the mechanical strength and vasoactive responsiveness of blood vessels [10]. Therefore, an increasing number of contractile smooth muscle cells impart blood vessels with good contractile function.

### 2.6. Extracellular Matrix Secretion

**Figure 6:**
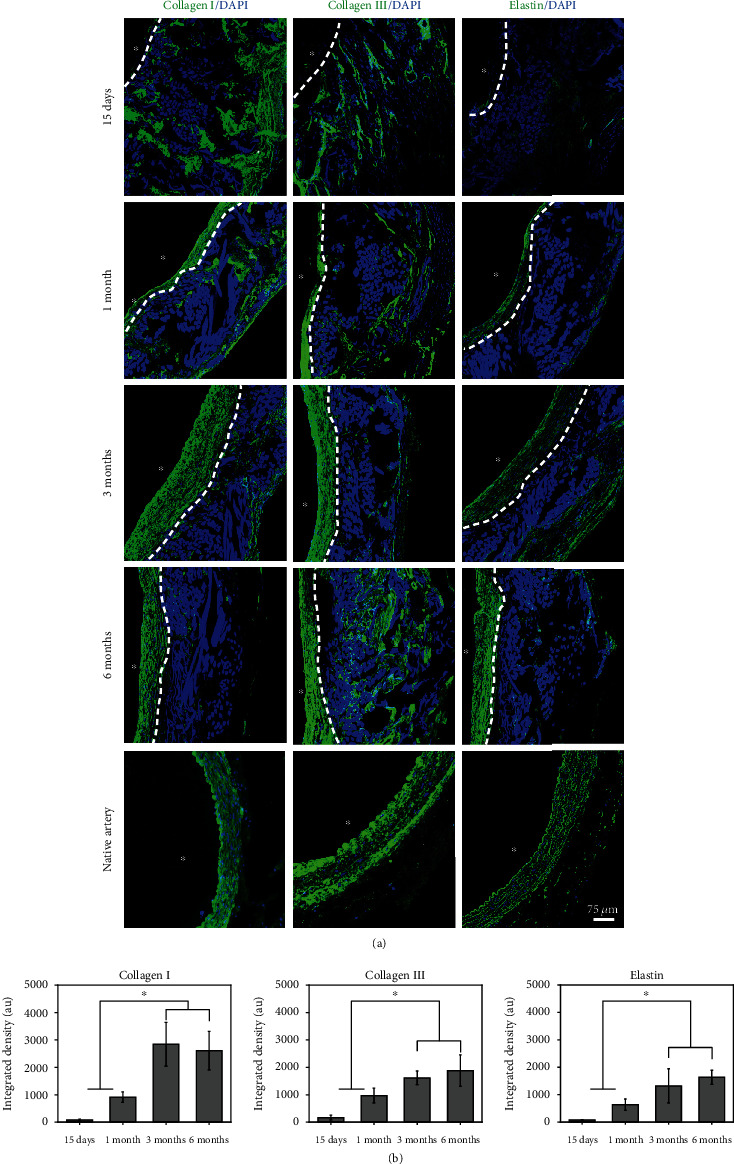
ECM deposition in the regenerated grafts over time. (a) Immunofluorescent staining showed that a small amount of collagens I and III (green) was visible near the lumen. Elastin antibody staining showed a low amount of elastin expression mostly near the lumen. Lumen is marked with ^∗^. The white dotted line denoted the interface between neotissue and the original graft. (b) Quantification of fluorescence intensity of collagen I, collagen III, and elastin in the neointima of each graft over time. ^∗^*p* < 0.05. Data are presented as the mean ± SD (*n* = 3). The fluorescence intensity in the neointima of the regenerated grafts at 15 days was defined as 100%.

To investigate the secretion and distribution of extracellular matrix (ECM) in the medium-porosity vascular grafts after implantation, collagen and elastin were observed by immunofluorescent staining.  Figure 6(a) shows that type III collagen and type I collagen were mainly distributed outside the grafts at 15 days. With increasing time, the collagen inside the grafts gradually increased. The cells began to express elastin at 1 month and expressed more elastin at 6 months (Figures 6(a) and 6(b)). In addition, a limitation of tissue-engineered vascular graft is that it cannot provide sufficient blood supply in the initial phase after implantation. Insufficient vascularization can result in improper cell integration or cell death in tissue-engineered constructs [42]. Figure S8 shows the formation of new capillaries in the outer layer of the grafts, which could deliver nutrients to the cells within the tissue.

### 2.7. Alignment of VSMC and ECM Organization

**Figure 7:**
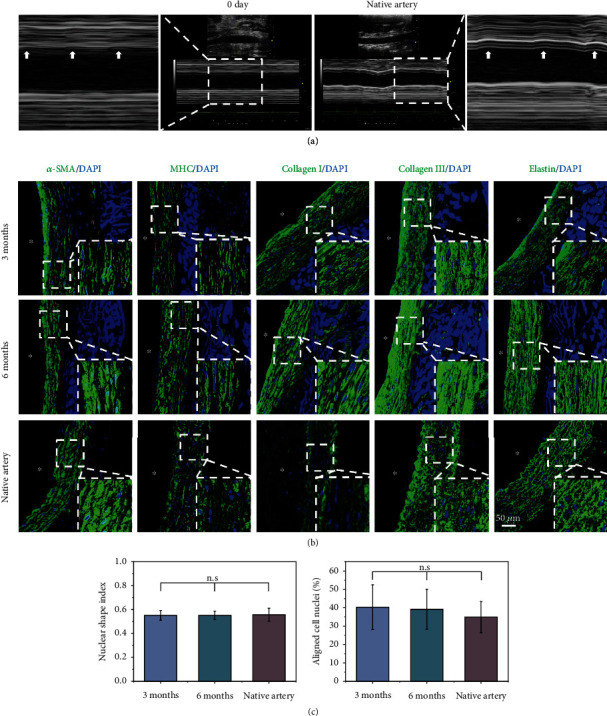
VSMC regeneration and ECM deposition with circumferential orientation *in vivo*. (a) M-mode color Doppler images of vascular grafts 0 days after implantation and the native arteries. (b) VSMC regeneration with circumferential orientation and ECM organization within the graft. (c) NSI and percentage of aligned cells of the native artery and the regenerated grafts. Data are presented as the mean ± SD (*n* = 4). “n.s.” means no significance. Arrows indicate the movement of the inner diameter of regenerated grafts and native artery, which suggests that regenerated grafts pulse synchronously with the native artery. Lumen is marked with ^∗^.

The vascular grafts with medium porosity showed obvious pulsation after implantation, and they pulsed synchronously with the native artery, which confirmed excellent compliance and closely matched native arterial compliance (Figure 7(a)).

The regenerated VSMCs were aligned in the circumferential direction in the vascular grafts (Figure 7(b)). The NSIs of VSMCs at 3 months and 6 months were 0.55 ± 0.04 and 0.55 ± 0.35, respectively, which was similar to that of the native artery (0.56 ± 0.53) (Figure 7(c)). The percentages of aligned VSMCs at 3 and 6 months and the native artery were 40.30 ± 12.19%, 39.18 ± 10.85%, and 34.95 ± 8.49, respectively, which also indicated that there was no obvious difference between the regenerated grafts and the native artery (Figure 7(c)). These data indicated that matching compliance between the medium porosity grafts and the native artery drives circumferential alignment of VSMCs.

In addition, Figure 7(b) shows that the extracellular matrix grew in the circumferential direction. It is worth noting that the regenerated grafts demonstrated high levels of continuous elastin (Figures 6 and 7(b)). Most tissue-engineered vascular grafts, whether made of acellular matrix or synthetic polymers, have difficulty triggering the production of elastin [7, 43]. Circumferential tension associated with pulsation after implantation could be the reason for increased elastin density in our study [44, 45].

### 2.8. Long-Term Evaluation of Braided Vascular Grafts

**Figure 8:**
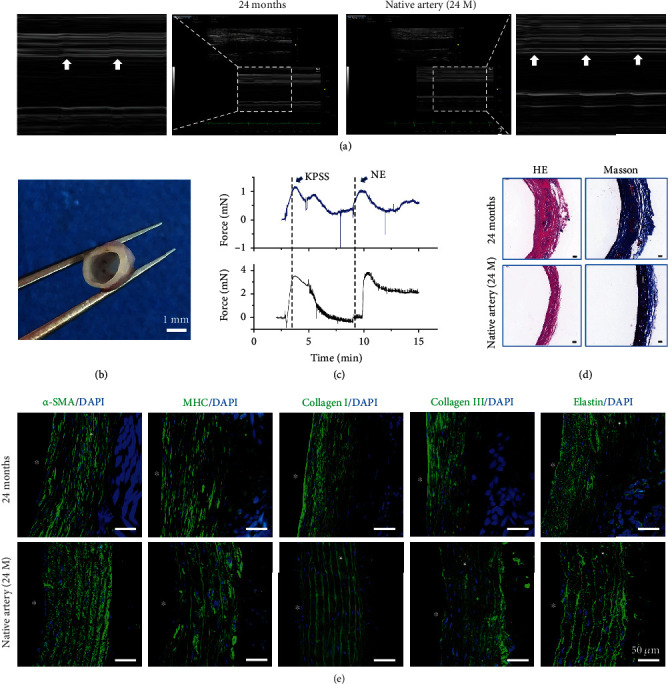
The compliant medium porosity vascular grafts displayed contractile function 2 years postimplantation. (a) M-mode color Doppler images of vascular grafts 2 years after implantation and native artery. (b) Image of the regenerated vascular grafts 2 years after implantation. (c) Representative concentration-response curves of grafts and native arteries to vasoconstrictors. The examination of physiological functions of the grafts displayed a certain extent of sensitivity to vasoconstrictors and grafts demonstrated constriction in response to both high potassium physiological saline solution (KPSS) and norepinephrine (NE). (d) HE and Masson's trichrome staining of the regenerated vascular grafts 2 years after implantation. (e) VSMC regeneration and ECM deposition in the regenerated grafts 2 years after implantation. The arrow indicates the movement of the inner diameter of regenerated grafts and the native artery, which suggests that regenerated grafts pulse synchronously with the native artery. Lumen is marked with ^∗^. Scale bar: (b) 1 mm, (d) 50 *μ*m, and (e) 50 *μ*m.

To investigate the long-term mechanical retention of the medium-porosity grafts, the regenerated grafts after 2 years of implantation were also evaluated. Four rats were used for the 2-year evaluation, and the patency rate was 75% (3/4). As observed in Doppler ultrasound, the neoarteries pulsed synchronously with the host artery after 2 years of implantation, indicating that the compliance of the regenerated grafts still matched that of the host artery (Figure 8(a) and Supplementary Video 1). More importantly, the generated media layer of neoarteries could still respond to vasomotor agonists after 2 years (Figures 8(b) and 8(c)).

Histological staining and immunofluorescent staining were used to investigate the organization of VSMCs and ECM in the neoarteries after 2 years of implantation. Immunofluorescent staining for VSMC and ECM organization showed that VSMCs and ECM were still circumferentially aligned (Figures 8(d) and 8(e)).

After 6 months, the silk fibroin sponge was almost degraded. As expected, the braided silk fibers remained visible throughout the entire implantation period even after 24 months (Figure S9).

## 3. Discussion

It is still a challenge to fabricate small-diameter vascular grafts mainly due to poor hemocompatibility and the mismatched mechanical properties of vascular grafts. Thrombosis, hyperplasia, and vascular blockage are usually due to the mismatch in compliance between the implanted vascular grafts and natural blood vessels, and these phenomena are a major reason for the failure of small-diameter vascular grafts [46]. The ideal vascular graft should not only have enough mechanical strength to withstand blood flow but also match the compliance of the native artery. The elastic moduli of the synthetic materials were in the range of GPa and MPa, which led to high burst pressures but low compliance [46–48]. More studies are needed to find the right balance between stiffness and compliance. Thus, the porosity of braided tubes was optimized in this study to improve the compliance and *in vivo* performance of vascular grafts.

In this study, the data suggested that the porosity of braided tubes could affect the mechanical properties of the vascular grafts. The graft with a higher porosity featured a higher elastic modulus and compliance but lower suture retention strength. Although compliance could be improved by increasing the porosity, the risk of surgery and the possibility of long-term mechanical failure then increase due to the lower suture retention strength. Future studies can use these models and data to design vascular grafts with braided structures.

Our *in vivo* results indicated that the porosity regulated blood leakage, cell infiltration, and neointima formation. High porosity could easily cause blood leakage, while low porosity blocked cell infiltration and often induced intimal hyperplasia. Cell infiltration plays a key role in vascular regeneration, including ECM secretion and blood vessel formation [49]. Poor cellularization usually hinders long-term vascular remodeling [50, 51]. Our results indicate that optimizing the porosity of the braided tubes could allow cells to easily infiltrate into the grafts. Macrophages play a key role in the development of stenosis and neovessel formation [52, 53]. Additionally, the M2 phenotype plays a positive role in vascular regeneration [35]. In our study, vascular grafts with medium porosity induced more M2 macrophages than vascular grafts with low porosity, which could result in different remodeling processes. While porosity is the main variable controlled here, changes in porosity can also alter the mechanical properties of the graft. It is possible that the interplay between these two properties influenced biological outcomes, especially macrophage polarization [54]. Consistent with Wang et al.'s study [35], CD68^+^ cells progressively migrated to the outer wall of the vascular grafts. The main phenotypes and changes of macrophages during vascular regeneration are still unclear, but we speculate that macrophages may stimulate cell migration into the grafts and modulate the differentiation and proliferation of smooth muscle cells. As time increased, the regeneration of smooth muscle cells was complete, and macrophages gradually migrated to the outer wall of the grafts.

Although various methods have been used to arrange cells in an orderly manner *in vitro*, such as mechanical loading, surface chemical treatment, and topographical patterning [55], it is still challenging to induce cell alignment in three-dimensional constructs. Moreover, it has been demonstrated that when using only the host's remodeling ability, circumferentially oriented VSMC layers are difficult to regenerate *in vivo* [34]. In this work, we fabricated vascular grafts with compliance mimicking native arteries, and our data indicated that circumferentially oriented VSMCs in medium-porosity vascular grafts were successfully regenerated after implantation as early as 3 months. By 3 months, the regenerated ECM arrangement was similar to that of the native artery. The VSMCs and ECM in the silk fibroin grafts appear to be more organized than those in previous studies at the same time point [29, 56]. In Zhu et al.'s study, the grafts had no obvious pulsation before 4 months, and by 12 months, the regenerated ECM was aligned [33]. We propose that one reason for the circumferential arrangement of VSMCs and ECM in this study may be the compliance mimicking native arteries of the medium-porosity grafts. The compliance mimicking native arteries of the medium-porosity grafts could allow smooth muscle cells to be subjected to cyclic stretching since the grafts showed obvious pulsation once implanted [57].

Vascular media remodeling is vital for successful vascular regeneration, and the SMC layer is mainly responsible for the mechanical strength and vasoactive response of the neoartery [10, 12]. In Li et al.'s study, only one regenerated graft showed slight contractile functions due to the stiffness of the PCL fibers after 18 months. These authors found that the number of VSMCs in the middle part of the graft wall decreased at 18 months. However, the VSMC numbers near the anastomosis sites had no apparent change. Indeed, due to the stimulating effect of the contraction and relaxation of the adjacent natural artery, the anastomotic site of the graft receives a slight pulsation [12]. Thus, it is important to maintain long-term vascular graft compliance to avoid degeneration of the VSMCs. VSMCs and extracellular matrix (ECM) are circumferentially aligned to allow the artery to contract [55, 58]. In this study, the regenerated neoarteries after 2 years showed a notable contractile function in response to vasoactive agents. This was mainly because the VSMCs obtained continuous pulsation due to the long-term compliance of the vascular grafts. However, there are few reports that grafts can maintain compliance matching for a long time *in vivo* [59].

The degradability of biomaterials is very important for tissue regeneration. If the vascular graft degrades too fast, rupture or aneurysm formation may occur [60]. Tissue regeneration may be inhibited by slow degradation and slow degradation may result in long-term inflammatory [61]. Therefore, the degradation rate of silk grafts should be adjusted to match the new tissue regeneration [62, 63]. It has been demonstrated that the *in vivo* implantation of the silk microtubes resulted in slower degradation rates than that of *in vitro* studies [64, 65]. Silk can be proteolytically degraded and resorbed *in vivo* over a longer period [66, 67]. Silk fibers generally lose most of their tensile strength within one year and become unrecognized at the site for more than two years or even longer *in vivo* [68]. In our study, we found that the braided silk fibers remained visible even after 2 years; these fibers may play a role in maintaining long-term compliance. Studies have shown that the degradability of silk fibers could be altered by degumming conditions and could be controlled to match the diverse needs of specific tissue regeneration requirements [61, 69]. Future studies may focus on adjusting the degradation rate of the braided silk fibers to match the new vascular regeneration.

Although medium-porosity vascular grafts greatly promoted the regeneration of circumferential VSMCs, and the regenerated neoartery showed obvious vasoconstriction function, this is only a study of the small animal model. Given that the vascular regeneration capacity of large animals and humans is slower than that of small animals [49, 70], experiments need to be carried out in large animal models to further evaluate the long-term patency and degradability of this medium-porosity vascular grafts. Further studies to determine the involvement of specific cell types and their origins during vascular regeneration would be beneficial. Vascular grafts are needed to specifically design to recruit these cell types to promote vascular regeneration. In addition, patients who need small-diameter vascular grafts usually suffer from diseases such as diabetes or atherosclerosis [71–73]; so, we need to further study the inflammatory status of braided vascular grafts in these disease states. Therefore, further optimization of the structure and composition of braided vascular grafts is crucial for clinical application.

## 4. Conclusions

The goal of this study was to optimize the porosity of braided tubes to improve the mechanical properties and *in vivo* performance of vascular grafts. Low-, medium-, and high-porosity braided silk tubes with sulfated silk fibroin and silk fibroin sponges were fabricated. The mechanical performance demonstrated that the graft with a higher porosity featured a higher elastic modulus and compliance but a lower suture retention strength. *In vivo* implantation indicated that the porosity regulated blood leakage, cell infiltration, and neointima formation. Compliant medium-porosity vascular grafts could induce the regenerated VSMCs to arrange in the circumferential direction as early as 3 months. In addition, the medium-porosity vascular grafts after 2 years of implantation showed potent vasoconstriction in response to vasomotor agonists, demonstrating the contractile function of the VSMCs.

## 5. Materials and Methods

### 5.1. Mathematical Model and Finite Element Analysis

To evaluate the effect of the porosity of the braided tubes on the compliance of vascular grafts, a finite element (FE) model was established for FE simulation analysis. SolidWorks (Dassault Systèmes, USA) was used to design a hollow cylinder unit composed of braided silk fiber tubes and sponges. The diameter and wall thickness of the conduit were 2 mm and 0.5 mm, respectively. Braided tubes made of 40 fibers were embedded in the middle position of the vascular graft wall (Figure 1(b)). The porosity of the grafts could be adjusted by changing the angle between the fibers. As shown in Figure 1(a), three vascular graft models were developed with different porosities (low, medium, and high). Figure 1(b) shows the model of the braided silk fibroin vascular grafts and the loading conditions used in the simulation. Uniform pressures of 80 mmHg and 120 mmHg were applied to the vascular grafts. The elastic modulus of the vascular base was set to 0.628 MPa [38], and the elastic modulus of the silk fiber was set to 289 MPa, as measured by the tensile test (Figure S10). The compliance was calculated as the change in vessel diameter with pressure. All numerical simulations and postprocessing were performed using the commercial package Abaqus (Dassault Systèmes, France).

### 5.2. Preparation of the Sulfated Silk Fibroin Solution

Silk fibers were purchased from Rugao Chunqiu Textile Co., Ltd. (Jiangsu, China). 5 g of silk fibers was boiled in an aqueous solution of 0.02 M Na_2_CO_3_ three times to remove sericin. The treated silk fibers were dissolved in 10 M LiBr (Sigma) at 60°C and put in a dialysis tube (Mw = 3500 Da, Pierce Chemical, Co. USA) in distilled water for 3 days to generate a silk fibroin solution. Silk fibroin sponges were obtained using a freeze dryer.

According to a previous method [37], 10 mL of chlorosulfonic acid was slowly added to 60 mL of pyridine (Sigma–Aldrich, USA). 10 g of silk fibroin sponges was added to this mixture, and the temperature was gradually increased to 80°C and kept at this temperature under stirring for 1 h. Then, 100 mL of distilled water was added to stop the reaction, and a sodium hydroxide solution was added to neutralize the mixture. 500 mL ethanol was added to precipitate soluble sulfated silk fibroin. The obtained sulfated silk fibroin was redissolved in water and dialyzed with water to generate a sulfated silk fibroin solution.

### 5.3. Fabrication of the Braided Silk Fibroin Vascular Grafts

Silk fibers with a diameter of 0.075 mm (Jiaxing Huayi Stock Co., Ltd, Jiaxing, China) were braided with a 40-bobbin braiding machine to form a tube with the inner diameter of 2 mm. Braided tubes with low, medium, and high porosity (Figure 2(a)) were designed by adjusting braiding angles (50°, 35°, and 20°). The braided silk tubes were treated with 0.02 M Na_2_CO_3_ solution to remove the sericin proteins. The braided tube (length 30 mm) was placed on a steel rod (ID 2 mm) and then inserted into a pipe (ID 3 mm, length 50 mm). Sulfated silk fibroin and silk fibroin solution (ratio = 1 : 1) were mixed and then added into the space between the steel rod and the pipe. The concentration of the sulfated silk fibroin and silk fibroin solutions was 5% (w/v), and the ratio of sulfated silk fibroin and silk fibroin to poly(ethylene glycol) diglycidyl ether (PGDE) (Sigma, USA) was 1 : 1. The mold was held at -20°C for 24 h, and then the grafts were removed from the rod and pipe. Then, the grafts were placed in water to remove the PGDE. The middle layer of the grafts was braided silk fibers, and the internal and external layers were porous silk fibroin sponges.

### 5.4. Morphological Analysis

The microstructure of the grafts was characterized using a scanning electron microscope (SEM) (Quanta™ 250 FEG, FEI) after sputtering with gold. The pore size and thickness of the graft were obtained from the SEM images with ImageJ software. Three samples were used, and 10 pictures were used for each sample.

### 5.5. Mechanical Properties

The compliance was calculated according to ISO-7198-1988. As shown in Figure 2(b), the pressure applied to the graft was adjusted by changing the height of the water tank. The change in graft diameter with internal pressure was recorded using a laser sensor (Keyence LS 7001). Grafts with an inner diameter of 2 mm and a length of 30–40 mm were used. The middle part of the samples was used to calculate the pressurized diameter at 80 and 120 mmHg. Compliance values were calculated with the following equation:

(1) $$\begin{array}{c} \%\text{compliance}=\frac{\left({\text{D}}_{P2}-{\text{D}}_{P1}\right)/{\text{RD}}_{P1}}{P2-P1}\times 10^{4}. \end{array}$$

*D*_*p*1_ and *D*_*p*2_ are the diameters at 80 and 120 mmHg, respectively.

The suture retention strength was determined with a mechanical tester (CARE Measurement & Control Corporation, IPBF-300, Tianjin, China). A 5-0 suture (Jin Huan, Shanghai, China) was inserted 2 mm from the edge of the grafts, and the suture was pulled at a speed of 3 mm/min until pulled out. At least 3 samples from each group were used.

A mechanical tester (CARE Measurement & Control Corporation, IPBF-300, Tianjin, China) was used to measure the longitudinal and circumferential tensile properties of the vascular grafts. Grafts with an inner diameter of 2 mm and a length of 30–40 mm were used for the longitudinal tensile properties test. Grafts with an inner diameter of 2 mm and a length of 5 mm were used for the circumferential tensile properties test. The grafts were loaded to failure at a speed of 3 mm/min. The maximum strain was calculated, and the slope of the stress-strain curve in the elastic region was calculated to obtain the elastic modulus of the vascular grafts.

### 5.6. In Vivo Implantation

A rat model of abdominal aorta replacement was established according to a previous protocol [35]. All animal experiments were approved by the Committee on Animal Care and Use at Beihang University following the Guidelines for the Care and Use of Laboratory Animals. Male Sprague–Dawley rats (280–300 g) were used in this study and anesthetized with 300 mg/kg chloral hydrate. Anticoagulation treatment was performed by using heparin (100 units/kg) before surgery. A blunt instrument was used to isolate the aorta. Clamps halted blood flow, and the aorta was transected. Vascular grafts 10 mm in length and 2 mm in diameter were sterilized using an autoclave before surgery. Grafts were then connected to the native artery using 9-0 nylon sutures (Jin Huan, Shanghai, China) via an end-to-end anastomosis. Anticoagulation and antiplatelet treatment was not performed after surgery. The patency of the grafts at different time points was assessed by Doppler ultrasound (Vevo 2100 System, Canada). The luminal diameter of the regenerated vascular grafts at different time points was investigated by ultrasound measurement. The detailed information of the statistical analysis of luminal diameter is available in the supplementary materials.

Five rats were used for the low-porosity group, 5 rats were used for the medium-porosity group, and 3 rats were used for the high-porosity group. Rats were retrieved at either 15 days or 1 month.

Due to the high mortality and low patency of high- and low-porosity vascular grafts, only the medium-porosity vascular grafts were used for different time point evaluations. Medium-porosity vascular grafts were implanted for 15 days and 1, 3, 6, and 24 months. 28 rats were used to evaluate the performance of the medium-porosity vascular grafts.

The rats were sacrificed at different time points. The grafts were harvested at each time point and were cut radially from the middle. One part of the grafts was embedded in optimal cutting temperature (OCT) compound (Tissue Tek) for frozen cross-section. Another part was longitudinally cut into two pieces. One piece of them was fixed in 2.5% glutaraldehyde for SEM observation. The other piece was used for gene expression analysis by real-time RT–PCR. The detailed procedure of SEM is available in the supplementary materials.

### 5.7. Quantitative Real-Time Reverse Transcription Polymerase Chain Reaction (RT–PCR) Analysis

Quantitative real-time RT–PCR was used to semiquantify M1 and M2 macrophage markers (iNOS and CD206) and smooth muscle cell markers (MHC). At 15 days, 1 month, and 3 months after implantation, mRNA was harvested using TRIzol Reagent (Invitrogen, Camarillo, CA). RNA was subsequently reverse-transcribed with a PrimeScriptTM RT Reagent Kit (Takara Bio, Otsu, Japan). SYBR Green Master Mix (Takara Bio, Otsu, Japan) was prepared, and real-time PCR was performed on iQ5 Multicolor Real-Time PCR Detection System (Bio-Rad, Hercules, CA). The comparative 2^-*ΔΔ*Ct^ method was used to calculate the relative expression of genes [74]. Primer sequences are listed in supplementary materials.

### 5.8. Histological Analysis

For histological staining, 4% paraformaldehyde was used to fix the samples. The harvested samples were cut longitudinally and fixed with 4% paraformaldehyde for endothelization analysis.

For histological analysis, samples were embedded in OCT, frozen in liquid nitrogen, and cut into 8 *μ*m thick sections. Hematoxylin and eosin (H&E) and Masson's trichrome staining were performed. For immunofluorescent staining, the frozen sections were washed with PBS, and 0.5% Triton X-100 solution was added. Samples were then incubated with 5% normal goat serums (Zhongshan, Beijing, China) for 1 hour at room temperature. Primary antibodies in goat serum were added to the samples and incubated overnight at 4°C. The primary antibodies used in this study were CD31 (Abcam, 1 : 50), *α*-SMA (Abcam, 1 : 200), smooth muscle myosin heavy chain I (MHC, Santa Cruz, 1 : 200), collagen type-I (collagen I, Abcam, 1 : 100), collagen type-III (collagen III, Abcam, 1 : 100), elastin (Abcam, 1 : 100), CD68 (Abcam, 1 : 100), and CD206 (Abcam, 1 : 100). Sections were incubated with a secondary antibody (Invitrogen, 1 : 200) for 1 hour at room temperature. 4,6-Diamidino-2-phenylindole (DAPI) (Southern Biotech, England) was used to stain the nuclei. The sections incubated with goat serum without primary antibodies were used as negative controls. Images were viewed under an Olympus light microscope (BX51; Olympus, Japan) and a confocal microscope (Leica, Germany). ImageJ software was used to measure the thickness of the neointima.

The percentage of antibody-labeled cells (CD68^+^ macrophages and CD206^+^ macrophages) was calculated as the ratio of fluorescent-labeled cells to all cells in the view. The total cell number within the grafts wall was calculated based on DAPI staining.

The fluorescence intensities of collagen I, collagen III, and elastin in the neointima of the regenerated grafts were analyzed using ImageJ software by outlining the neointima of the regenerated grafts and measuring the fluorescence intensity.

The perimeter (*L*) and spreading area (*S*) of the nuclei at 3 months and 6 months were calculated, and nuclear shape index (NSI) was calculated using the following formula: 4*πS*/*L*^2^. The NSI of nuclei with nearly circular shapes is close to 1, while the NSI of nuclei with an elongated morphology approaches 0 [34]. The detailed information of the statistical analysis of neointima thickness, CD68^+^ and CD206^+^ macrophage percentage, relative quantification of fluorescence intensity of collagen I, collagen III, and elastin, and nuclei shape analysis is also available in the supplementary materials.

### 5.9. Vascular Function Assessment

The vasomotor properties of the grafts 2 years after implantation were assessed by vascular tension myography [75]. Graft rings close to the anastomoses (3 mm in length) were harvested and fixed on a wire mygraph system (DMT 620 M, Denmark) at 37°C. The contractile responses were induced by high potassium physiological saline solution (KPSS) (composition in mM: KCl 60, NaCl 74.7, MgSO_4_·7H_2_O 1.17, KH_2_PO_4_ 1.18, CaCl_2_ 1.16, EDTA 0.026, NaHCO_3_ 14.9, and glucose 5.5, pH 7.4) and norepinephrine (NE: 0.001 M). The vascular tension signal was recorded by specific software (PowerLab, ADInstruments).

### 5.10. Statistical Analysis

All the data are shown as the means ± standard deviations. Student's *t*-test was used for two-sample statistical comparison, while one-way analysis of variance (ANOVA) followed by Tukey's test was used for groups greater than two. A value of *p* < 0.05 was considered statistically significant.

## Data Availability

All data supporting the findings of this study are available within the paper and its supplementary information.

## Supplementary Materials

Supplementary Materials Figure S1: SEM images of the internal surface of medium-porosity vascular grafts. Scale bar: (a) 300 *μ*m and (b) 100 *μ*m. Figure S2: H&E cross-section staining of occluded low-porosity vascular grafts (2 weeks). Scale bar: 100 *μ*m. Figure S3: H&E staining and Masson's trichrome staining of the graft (day 0). Scale bar: 100 *μ*m. Figure S4: (a–d) the distribution and changes of CD68-positive cells in medium-porosity vascular grafts during the remodeling process. (e) mRNA expression levels of iNOS and CD206 at different time points. ^∗^*p* < 0.05. Data are presented as the mean ± SD (*n* = 3). Scale bar: (a, b) 500 *μ*m, (c) 75 *μ*m, and (d) 50 *μ*m. The blue color represents the silk scaffold (nonspecific binding) and nuclei, while the green color represents CD68. Figure S5: (a) time-dependent gross changes of the medium-porosity vascular grafts integrated with host artery. Microvessels and vascularization were noticed in the surrounding tissue of the grafts. (b) Representative lumen surface stereomicroscopic images of the regenerated vascular grafts at different time points. Scale bar: (a) 500 *μ*m and (b) 500 *μ*m. Figure S6: The morphology of the medium-porosity vascular grafts at 6 months was observed under stereomicroscope. Scale bar: 200 *μ*m. Figure S7: mRNA expression levels of MHC at different time points. ^∗^*p* < 0.05. Data are presented as the mean ± SD (*n* = 3). Figure S8: the new capillary formation on medium-porosity vascular grafts at different time points. Scale bar: 75 *μ*m. Figure S9: SEM images of the explanted grafts showed the presence of braided silk fibers after 3, 6, and 24 months. Scale bar: 100 *μ*m. Arrows indicate the braided silk fibers. Figure S10: representative stress-strain curves of different silk fibers. A mechanical tester (CARE Measurement & Control Corporation, IPBF-300, Tianjin, China) was used to measure the elastic modulus of silk fibers. The elastic modulus of the silk fibers was 289 ± 41 MPa, as measured by the tensile test (*n* = 4). Supplementary video 1: Doppler ultrasound showed neoarteries pulsed synchronously with the host artery after 2 years of implantation.
