# Supplementary material for: The Regulatory Effect of Braided Silk Fiber Skeletons with Differential Porosities on In Vivo Vascular Tissue Regeneration and Long-Term Patency
Source: Research (Wash D C). 2022 Nov 11;2022:9825237. doi: 10.34133/2022/9825237 (PMC9703915; doi:10.34133/2022/9825237)
Supplement: Supplementary Materials — Figure S1: SEM images of the internal surface of medium-porosity vascular grafts. Scale bar: (a) 300 μm and (b) 100 μm. Figure S2: H&E cross-section staining of occluded low-porosity vascular grafts (2 weeks). Scale bar: 100 μm. Figure S3: H&E staining and Masson's trichrome staining of the graft (day 0). Scale bar: 100 μm. Figure S4: (a–d) the distribution and changes of CD68-positive cells in medium-porosity vascular grafts during the remodeling process. (e) mRNA expression levels of iNOS and CD206 at different time points. ∗p < 0.05. Data are presented as the mean ± SD (n = 3). Scale bar: (a, b) 500 μm, (c) 75 μm, and (d) 50 μm. The blue color represents the silk scaffold (nonspecific binding) and nuclei, while the green color represents CD68. Figure S5: (a) time-dependent gross changes of the medium-porosity vascular grafts integrated with host artery. Microvessels and vascularization were noticed in the surrounding tissue of the grafts. (b) Representative lumen surface stereomicroscopic images of the regenerated vascular grafts at different time points. Scale bar: (a) 500 μm and (b) 500 μm. Figure S6: The morphology of the medium-porosity vascular grafts at 6 months was observed under stereomicroscope. Scale bar: 200 μm. Figure S7: mRNA expression levels of MHC at different time points. ∗p < 0.05. Data are presented as the mean ± SD (n = 3). Figure S8: the new capillary formation on medium-porosity vascular grafts at different time points. Scale bar: 75 μm. Figure S9: SEM images of the explanted grafts showed the presence of braided silk fibers after 3, 6, and 24 months. Scale bar: 100 μm. Arrows indicate the braided silk fibers. Figure S10: representative stress-strain curves of different silk fibers. A mechanical tester (CARE Measurement & Control Corporation, IPBF-300, Tianjin, China) was used to measure the elastic modulus of silk fibers. The elastic modulus of the silk fibers was 289 ± 41 MPa, as measured by the tensile test (n = 4). Supplementary video 1: [file 9825237.f1.zip › Supplementary Material - Clean version.docx]

**Supplementary Material**

**1. Method and materials**

**1.1 Measurement of luminal diameter of the regenerated grafts**

The luminal diameter of the regenerated vascular grafts at different time points was investigated by ultrasound measurement. Three points per sample and four samples per group were included to obtain the statistical results.

**1.2 SEM observation of regenerated endothelium**

The explanted grafts were rinsed with PBS, ﬁxed in 2.5% (v/v) glutaraldehyde for 12 h and dehydrated in ascending series of ethanol. After air-drying at room temperature, samples were affixed onto aluminum stubs with carbon tape and sputter-coated with gold. Then, the lumen of regenerated grafts was observed under SEM (Quanta™ 250 FEG, FEI).

**1.3 Quantitative real-time reverse transcription polymerase chain reaction (RT–PCR) analysis**

Primers (Shanghai, China) were synthesized as follows:

iNOS, 5'-CCT GGT GCA AGG GAT CTT GG-3' and 5'-GAG GGC TTG CCT GAG TGA GC-3', CD206, 5'-GGT TCC GGT TTG TGG AGC AG-3' and 5'-TCC GTT TGC ATT GCC CAG TA-3', and MHC: 5'-AAGCAGCTCAAGAGGCAG-3' and 5'-AAGGAACAAATGAAGCCTCGTT-3'.

**1.4** **Immunofluorescence analysis**

ImageJ software was used to measure the thickness of the neointima. For quantitative analysis of neointima thickness, three cross-sections images per section, three sections per sample and five samples per group were included to obtain the statistical results.

The percentage of antibody-labeled cells (CD68^+^ macrophages and CD206^+^ macrophages) was calculated as the ratio of fluorescent-labeled cells to all cells in the view. The total cell number within the grafts wall was calculated based on DAPI staining. For quantitative analysis of CD68^+^ and CD206^+^ macrophage percentage, four images per section, three sections per sample, and three samples per group were included to obtain the statistical results.

The fluorescence intensities of collagen I, collagen III, and elastin in the neointima of the regenerated grafts were analyzed using ImageJ software by outlining the neointima of the regenerated grafts and measuring the fluorescence intensity. For quantitative analysis of fluorescence intensity of collagen I, collagen III, and elastin in the neointima of the regenerated grafts, four images per section, three sections per sample, and three samples per group were included to obtain the statistical results.

The perimeter (L) and spreading area (S) of the nuclei at 3 months and 6 months were calculated, and nuclear shape index (NSI) was calculated using the following formula: 4ᴫS/L^2^. For nuclei shape analysis, three high-magnification images per section, three sections per sample, and four samples per group were included to obtain the statistical results.

**Supplementary Figures**


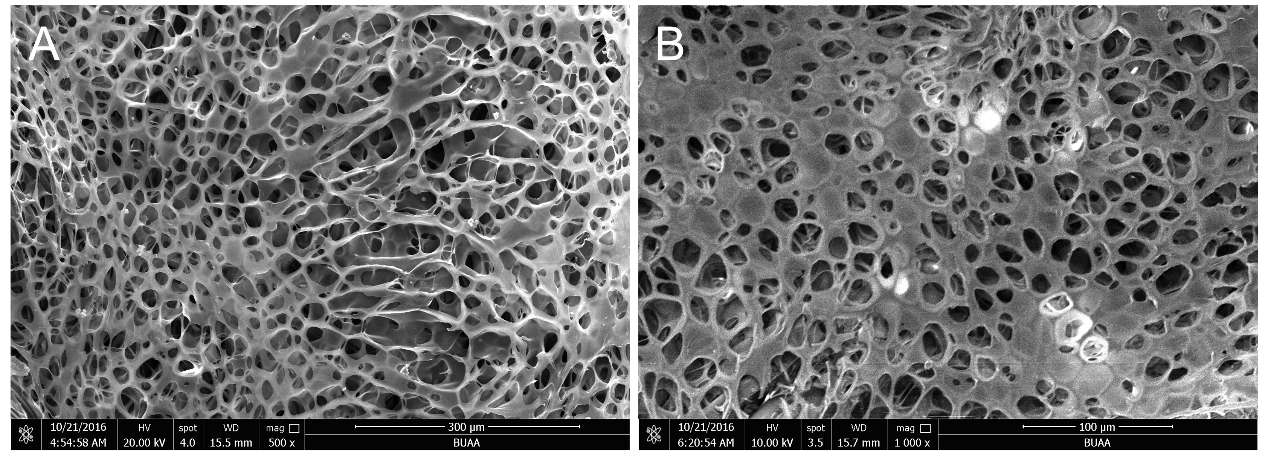


**Fig. S1**. SEM images of the internal surface of medium-porosity vascular grafts. Scale bar: A: 300 μm; B: 100 μm.


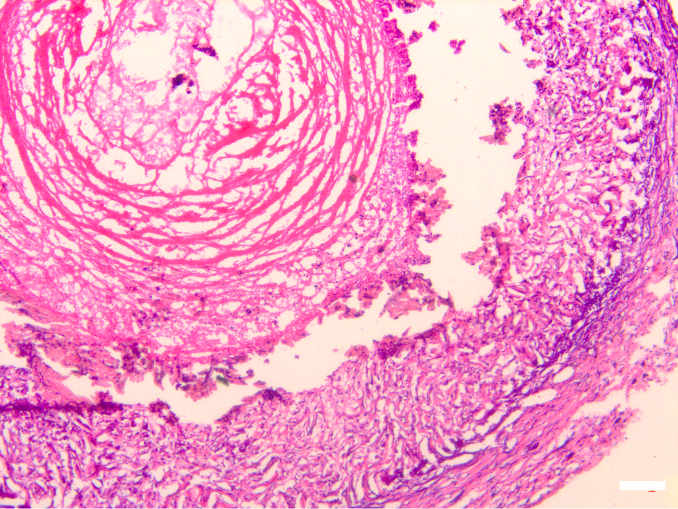


**Fig. S2.** H&E cross-section staining of occluded low-porosity vascular grafts (15 days). Scale bar: 100 μm.


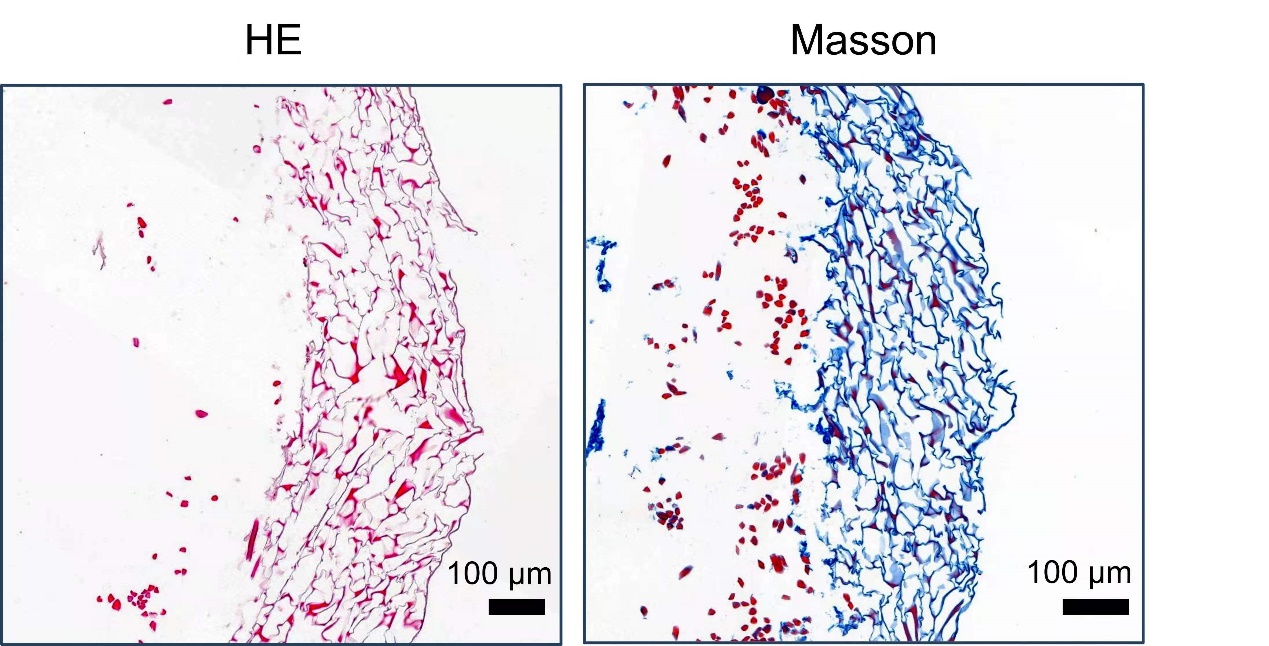


**Fig. S3.** H&E staining and Masson’s trichrome staining of the graft (day 0). Scale bar: 100 μm.


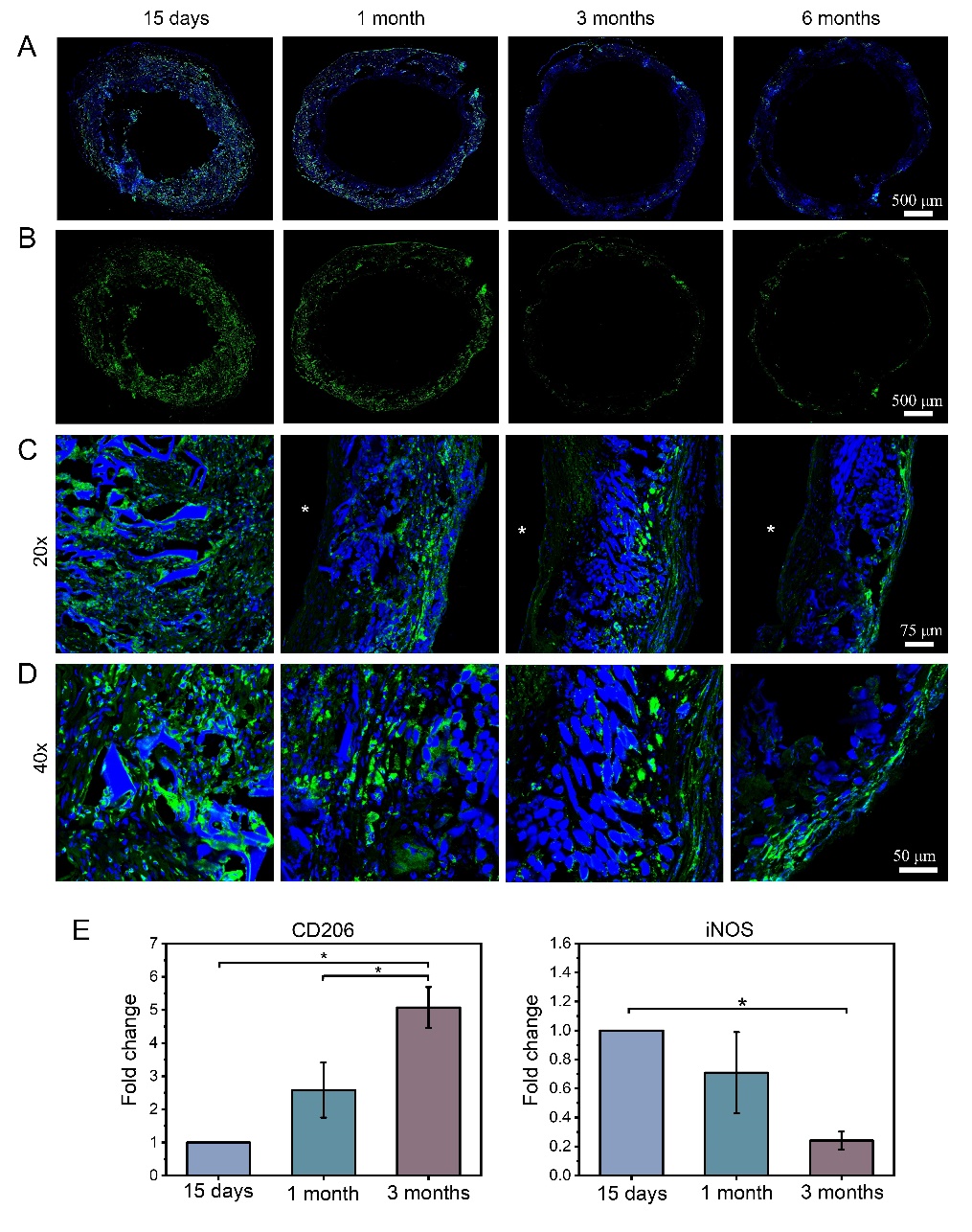


**Fig. S4.** A-D. The distribution and changes of CD68 positive cells in medium-porosity vascular grafts during the remodeling process. E. mRNA expression levels of iNOS and CD206 at different time points. **p* < 0.05. Data are presented as the mean ± SD (n = 3). Scale bar: A and B: 500 μm, C: 75 μm, D: 50 μm. The blue color represents the silk scaffold (non-specific binding) and nuclei, while the green color represents CD68.


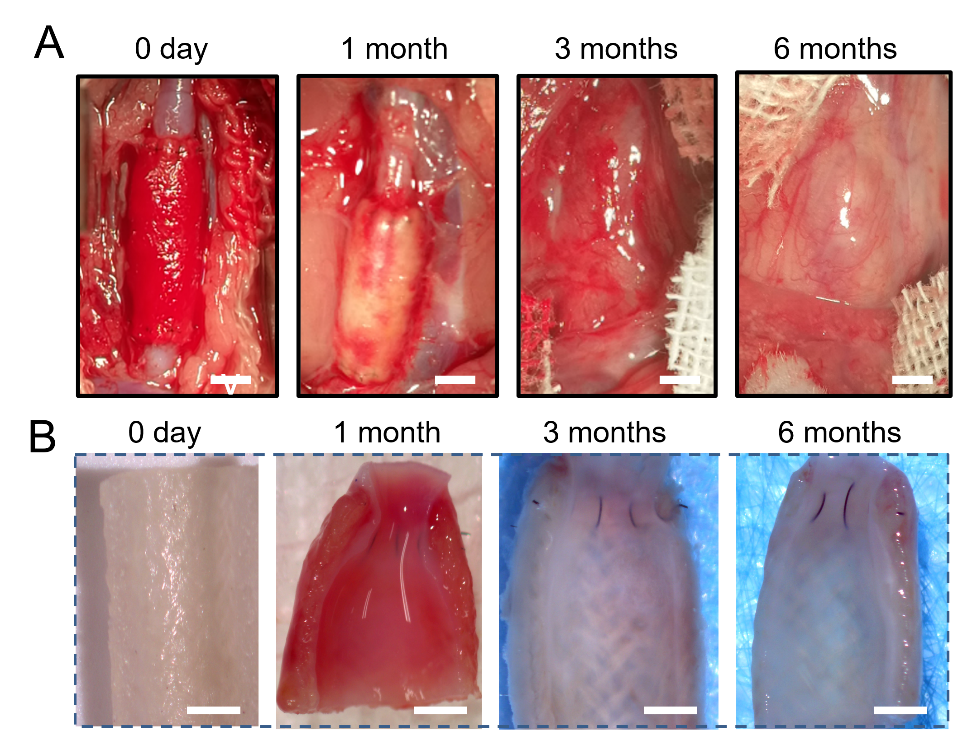


**Fig. S5.** A. Time-dependent gross changes of the medium-porosity vascular grafts integrated with host artery. Micro vessels and vascularization were noticed in the surrounding tissue of the grafts. B. Representative lumen surface stereomicroscopic images of the regenerated vascular grafts at different time points. Scale bar: A: 500 μm; B: 500 μm.


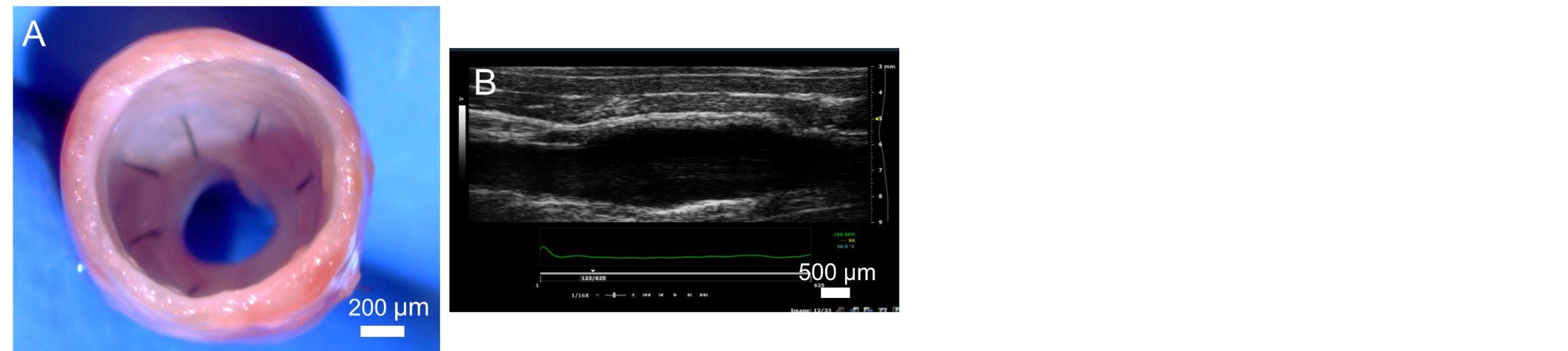


**Fig. S6.** The morphology of the medium-porosity vascular grafts at 6 months was observed under stereomicroscope. Scale bar: 200 μm.

**Fig. S7**. mRNA expression levels of MHC at different time points. **p* < 0.05. Data are presented as the mean ± SD (n = 3).


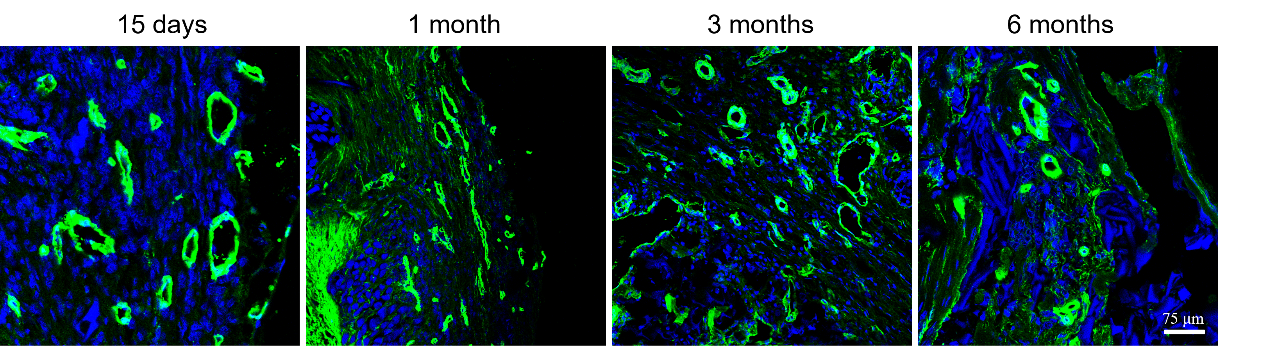


**Fig. S8**. The new capillary formation on medium-porosity vascular grafts at different time points. Scale bar: 75 μm.


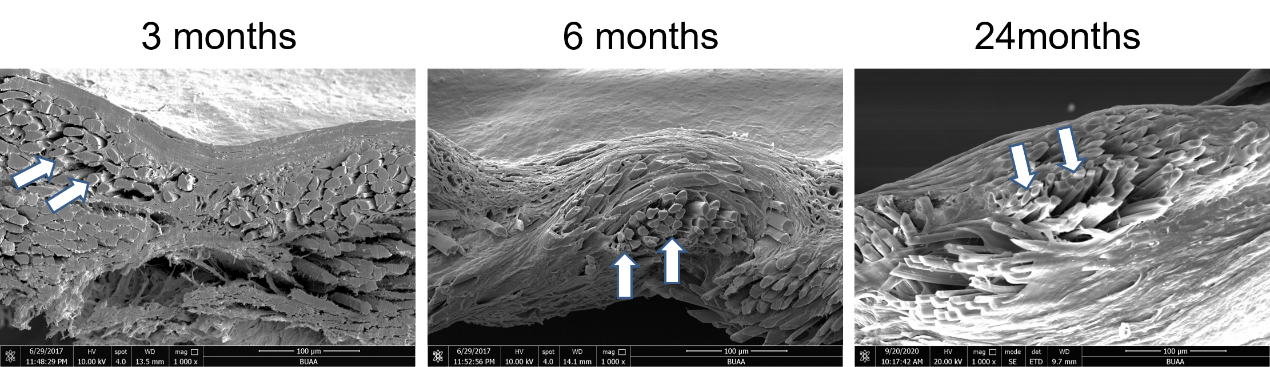


**Fig. S9**. SEM images of the explanted grafts showed that the presence of braided silk fibers after 3, 6, and 24 months. Scale bar: 100 μm. Arrows indicate the braided silk fibers.


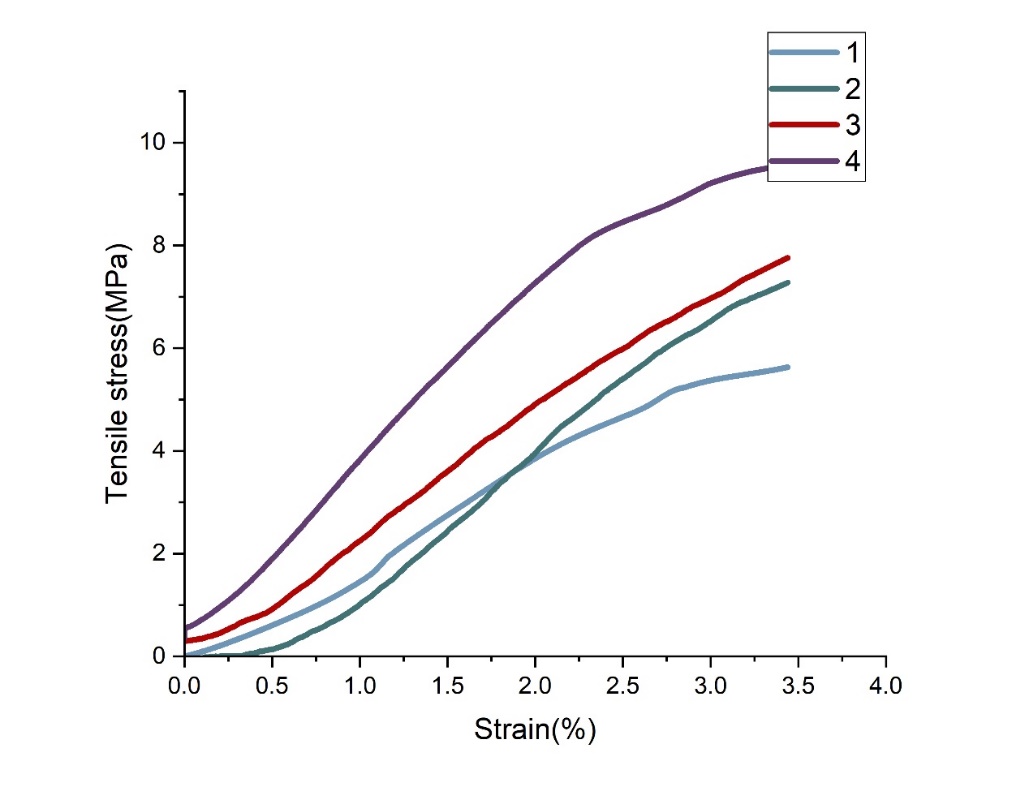


**Fig. S10**. Representative stress-strain curves of different silk fibers. A mechanical tester (CARE Measurement & Control Corporation, IPBF-300, Tianjin, China) was used to measure the elastic modulus of silk fibers. The elastic modulus of the silk fibers was 289 ± 41 MPa, as measured by the tensile test (n=4).

**Supplementary video 1**. Doppler ultrasound showed neo-arteries pulsed synchronously with the host artery after 2 years of implantation.
